# Determinants of *Plasmodium falciparum* multiplicity of infection and genetic diversity in Burkina Faso

**DOI:** 10.1186/s13071-020-04302-z

**Published:** 2020-08-20

**Authors:** Paul Sondo, Karim Derra, Toussaint Rouamba, Seydou Nakanabo Diallo, Paul Taconet, Adama Kazienga, Hamidou Ilboudo, Marc Christian Tahita, Innocent Valéa, Hermann Sorgho, Thierry Lefèvre, Halidou Tinto

**Affiliations:** 1 Institut de Recherche en Sciences de la Santé/Clinical Research Unit of Nanoro (IRSS-URCN), Nanoro, Burkina Faso; 2 Institut National de Santé Publique/Centre Muraz de Bobo-Dioulasso, Bobo-Dioulasso, Burkina Faso; 3 Centre de Recherche en Écologie et Évolution de la Santé (CREES), Montpellier, France; 4 Laboratoire Mixte International sur les Vecteurs (LAMIVECT), Bobo-Dioulasso, Burkina Faso; 5 Institut de Recherche pour le Développement (IRD), Centre National pour la Recherche Scientifique (CNRS), Maladies Infectieuses et Vecteurs: Ecologie, Génétique, Evolution et Contrôle (MIVEGEC), Université de Montpellier, Montpellier, France

**Keywords:** Malaria, *Plasmodium falciparum*, Multiplicity of infection, *msp1*, *msp2*

## Abstract

**Background:**

Investigating malaria transmission dynamics is essential to inform policy decision making. Whether multiplicity of infection (MOI) dynamic from individual infections could be a reliable malaria metric in high transmission settings with marked variation in seasons of malaria transmission has been poorly assessed. This study aimed at investigating factors driving *Plasmodium falciparum* MOI and genetic diversity in a hyperendemic area of Burkina Faso.

**Methods:**

Blood samples collected from a pharmacovigilance trial were used for polymerase chain reaction genotyping of the merozoite surface proteins 1 and 2. MOI was defined as the number of distinct parasite genotypes co-existing within a particular infection. Monthly rainfall data were obtained from satellite data of the Global Precipitation Measurement Database while monthly malaria incidence aggregated data were extracted from District Health Information Software 2 medical data of the Center-West health regional direction.

**Results:**

In the study area, infected people harboured an average of 2.732 (± 0.056) different parasite genotypes. A significant correlation between the monthly MOI and the monthly malaria incidence was observed, suggesting that MOI could be a good predictor of transmission intensity. A strong effect of season on MOI was observed, with infected patients harbouring higher number of parasite genotypes during the rainy season as compared to the dry season. There was a negative relationship between MOI and host age. In addition, MOI decreased with increasing parasite densities, suggesting that there was a within-host competition among co-infecting genetically distinct *P. falciparum* variants. Each allelic family of the *msp1* and *msp2* genes was present all year round with no significant monthly fluctuation.

**Conclusions:**

In high malaria endemic settings with marked variation in seasons of malaria transmission, MOI represents an appropriate malaria metric which provides useful information about the longitudinal changes in malaria transmission in a given area. Besides transmission season, patient age and parasite density are important factors to consider for better understanding of variations in MOI. All allelic families of *msp1* and *msp2* genes were found in both dry and rainy season. The approach offers the opportunity of translating genotyping data into relevant epidemiological information for malaria control.
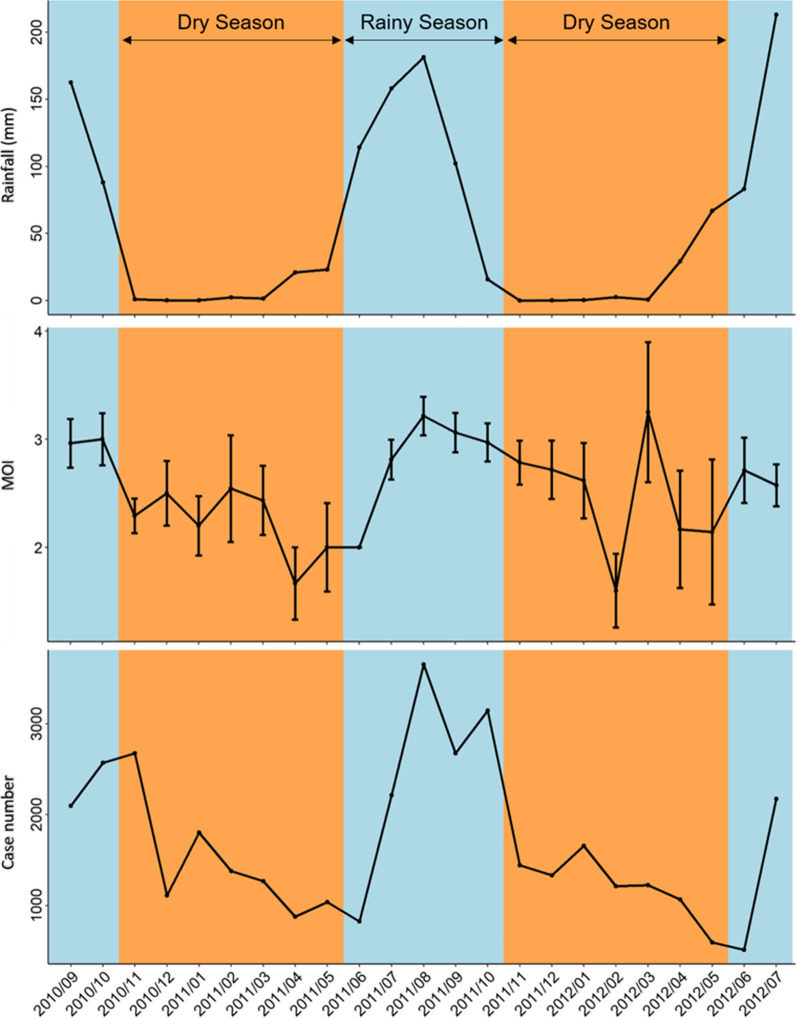

## Background

Malaria is the most common life-threatening disease and nearly half of the world’s population is exposed to malaria infection [1]. Each year, *Plasmodium falciparum*, the most prevalent and most dangerous *Plasmodium* species infects about 228 million people causing 400,000 deaths worldwide [1]. In Burkina Faso, malaria remains endemic with over 10 million clinical episodes and 4294 deaths annually [1]. In spite of the several control interventions that have been implemented such as seasonal malaria chemoprevention, distribution of treated mosquito nets, intermittent preventive treatment for pregnant women, the number of clinical episodes still remain high though a drop in the related mortality has been observed since 2015 [1]. Restricting the measurement of the effectiveness of malaria control interventions to variation of morbidity and related mortality would considerably underestimate their real impact. Factors such as the reporting system and health care attendance greatly affect the assessment of these indicators. For example, in Burkina Faso, the free healthcare policy for children under five years of age and pregnant women adopted by the government in 2016 increased the population attendance to public health services [2]. This partially explains the increasing number of malaria reported cases thereafter and the relatively low related mortality [2].

New malaria metrics are needed to complement the assessment of malaria transmission dynamics in a context of multiple control interventions. Multiplicity of infection (MOI), the number of different parasite genotypes co-existing within a particular infection, has been suggested as a useful malaria metric describing transmission dynamics [3–6]. Whether MOI dynamic from individual infections could be a reliable malaria metric in high transmission settings with marked variation in seasons of malaria transmission has been poorly assessed. Existing malaria genotype data in Burkina Faso are usually limited to particular time points which do not allow the assessment of the year-round trend of the MOI dynamic.

Furthermore, factors driving the MOI remain poorly understood with controversial findings though this elucidation is crucial to justify its epidemiological significance. For instance, MOI correlation with age and parasite density was reported either positively [7–9] or negatively [10, 11]. Similarly, it was commonly reported that host age influenced *P. falciparum* MOI but the directionality of the relationship was controversially reported [12, 13]. While several studies reported a negative correlation between the MOI and host age, underpinning the acquisition of an anti-parasite immunity in older individuals [10, 14], other studies reported a positive relationship [7, 15]. Another important factor which is most often ignored is the effect of sex although sex-mediated differences in parasite infections is known [16, 17]. A recent study conducted in Cameroon reported that the MOI value was marginally higher in male than in female participants [18], while a study in the Republic of the Congo reported no effect of patient sex on the MOI [15].

Finally, whether specific *P. falciparum* variants of the *msp1* and *msp2* allelic families are well adapted to specific seasonal characteristics of the study area than others, is hitherto unknown. In neighboring Ghana, where malaria transmission tends to be perennial, the relative frequencies of *msp1* allelic families were found to be dynamic across transmission season [19]. In Burkina Faso, despite marked seasonal variations, data exploring *P. falciparum* genetic diversity regarding this seasonal pattern are not available.

This study explored the influence of season, patient age, sex, and parasite density on both *P. falciparum* multiplicity of infection and genetic diversity in an endemic area of Burkina Faso with marked seasonality of malaria transmission.

## Methods

### Study site and source of samples

Clinical data were collected from September 2010 to October 2012 at two primary health centers (Nanoro and Nazoanga) of the Nanoro sanitary district in Burkina Faso. Nanoro is one of the sentinel sites for the assessment of antimalarial therapeutic efficacy and is located at 85 km from Ouagadougou, the capital city of Burkina Faso. Malaria is hyperendemic with seasonal variation in transmission which peaked between July/August and October/November. As in other sub-Sahara African countries, *P. falciparum* is the most common *Plasmodium* species. The population mostly practice subsistence farming with Mossi being the major ethnic group, and Gourounsi and Fulani representing minor groups.

Dried blood spots were collected from 724 malaria patients (aged from 6 months to 40 years-old: median: 4 years, interquartile range (IQR): 2–6, male: female ratio = 381:343) with parasite density ≥ 2000/µl, attending a pharmacovigilance trial over 23 months of the study period. For this investigation, only samples collected on day 0, i.e. from screened participants before the administration of study drugs artemether-lumefantrine (AL) or artesunate-amodiaquine (ASAQ) were considered. Screened participants included any patient attending the two health facilities during the study period with fever or history of fever or clinical suspicion of malaria according to the routine medical staff of the health facilities. Those who met all the criteria for the effectiveness study were included in this cohort and treated with either AL or ASAQ and those who failed were treated with ASAQ, the only available ACT during the study period in the drug stores of the health facilities. Details of the study site and sampling procedures were described previously elsewhere [14, 20, 21].

### Molecular analysis

Molecular analysis was performed at the Molecular Biology Laboratory of Centre Muraz of Bobo-Dioulasso, Burkina Faso, from September 2012 and October 2014. Genomic DNA was extracted from dry blood spots using the QIamp DNA Kit (Qiagen, Hilden, Germany) following the manufacturer’s procedure. A nested PCR amplification (using a Mastercycler® Gradient, (Eppendorf, Hamburg, Germany) and a Biometra thermal cycler (Analytik Jena, Jena, Germany) PCR machines of *msp1* block 2 and *msp2* block 3 was performed as previously described [14, 20]. Briefly, 5 µl of DNA extract serving as a DNA template were used to initiate the first msp PCR round. Following completion of the first PCR round, 1 µl of the PCR product of the first PCR round was used as DNA template to launch the 2nd round of nested PCR for *msp1* and *msp2* using family-specific primers. All PCR rounds were carried out with a total volume of reaction mixture of 25 µl. Fragments were revealed by ethidium bromide stained agarose gel electrophoresis using Labnet^TM^ (Labnet International, New York, USA) and Mupid^TM^-One (Nippon Genetics Europe, Dueren, Germany) electrophoresis machines. Gels were visualized by ultraviolet transillumination and band sizes were calculated using Photo Capt^MW^ (version 11.01).

### Determination of MOI and allelic frequency

MOI was defined as the number of distinct parasite genotypes co-existing within a given infection based on the genotyping of the *msp1* block 2 gene (K1, MAD20 and RO33) and the *msp2* block 3 gene (3D7 and FC27) [3, 7, 14, 22]. Each PCR round using family-specific primers can reveal several alleles differing in band size resulting from variation of repeat allotypes encoding a single amino-acid motif which characterize the family [23, 24]. For example, the PCR round using MAD20-specific primers can reveal two distinct bands indicating that two genotypes of the allelic family MAD 20 were present in this infection. Similarly, the other PCR rounds each using K1-, RO33-, 3D7- or FC27-specific primers could result e.g. in 2, 1, 3, and 1 band respectively. With this example, the genotyping of *msp1* revealed 2 (MAD20) + 2 (K1) + 1 (RO33) = 5 genetic variants, while the genotyping of *msp2* revealed 3 (3D7) + 1 (FC27) = 4 genetic variants. In other words, MOI for *msp1* for each clinical sample represented the number of PCR fragments obtained from K1 + MAD20 + RO33 gels, while the MOI for *msp2* represented the number of PCR fragments obtained from 3D7 + FC27 gels. Samples with a single parasite genotype at both *msp1* and *msp2* loci were classified as mono-infections while samples with more than one parasite genotype at any of the two loci were classified as multiple infections. The final MOI value for each clinical isolate represented the maximum MOI value from both *msp1* and *msp2* loci i.e. MOI = 3 if MOI_*msp1*_ = 3 and MOI_*msp2*_ =2.

The frequency of allelic family (K1, MAD20 and RO33 for *msp1*, and 3D7 and FC27 for *msp2*) was calculated as the ratio of the number of PCR bands obtained for each family to the overall number of gene-specific PCR bands obtained from PCR positive samples. For each patient, the number of fragments observed for a given allelic family was divided by the total number of fragments observed for the *msp1* or *msp2* genes. For example, if for a given blood sample, 5 K1 fragments, 1 MAD20 fragment and 0 RO33 fragments were observed, the relative frequencies of K1, MAD20 and R033 for this patient were respectively 0.83 (5/6), 0.17 (1/6) and 0 (0/6).

### Rainfall and malaria incidence data

Monthly rainfall data were obtained from satellite data of the Global Precipitation Measurement (GPM) Database [25]. Monthly rainfall data was used to make a distinction between the dry season that starts from November to May and the rainy season from June to October. Malaria incidence data were extracted from District Health Information Software 2 (DHIS2-Endos) medical data of the Center-West sanitary regional direction [26].

### Statistical analysis

All statistical analyses were performed with R (version 3.5.1) software [27]. A generalized linear model (GLM) with Poisson errors was used to investigate the effect of season (2 levels: dry *vs* rainy), sex (2 levels: male, female), parasite density, age and interactions, on *P. falciparum* multiplicity of infection. For each allelic family (K1, MAD20, RO33, 3D7 and FC27), a GLM with binomial errors was used to investigate the effect of time (numeric variable from 1 (September 2010) to 23 (July 2012)) on the relative frequency of the studied allelic family. A GLM with quasibinomial errors was used to explore the effect of season (2 levels: dry *vs* rainy), sex (2 levels), parasite density, age, and interactions on *P. falciparum* genetic diversity. Model simplification used stepwise removal of terms, followed by likelihood ratio tests (LRT). Term removals that significantly reduced explanatory power (*P* < 0.05) were retained in the minimal adequate model.

## Results

### Multiplicity of infection

A total of 724 samples were successfully genotyped. Of these, 316 (43.65%) samples were collected during the dry season while 408 (56.35%) were collected during the rainy season. In total, *msp1* yielded a total of 1717 PCR fragments ranging between 142–497 bp for the K1 allelic family, 91–319 bp for the MAD20 allelic family, 95–391 bp for the RO33 allelic family, while 1653 fragments ranging between 170–773 bp for the 3D7 allelic family and 180–719 bp for the FC27 allelic family were obtained from *msp2* genotyping. The MOI ranged from 1 to 7 parasite genotypes with a mean of 2.732 (± 0.056) different parasite genotypes co-existing per isolate.

### Monthly dynamics of MOI, malaria incidence, and rainfall

**Fig. 1:**
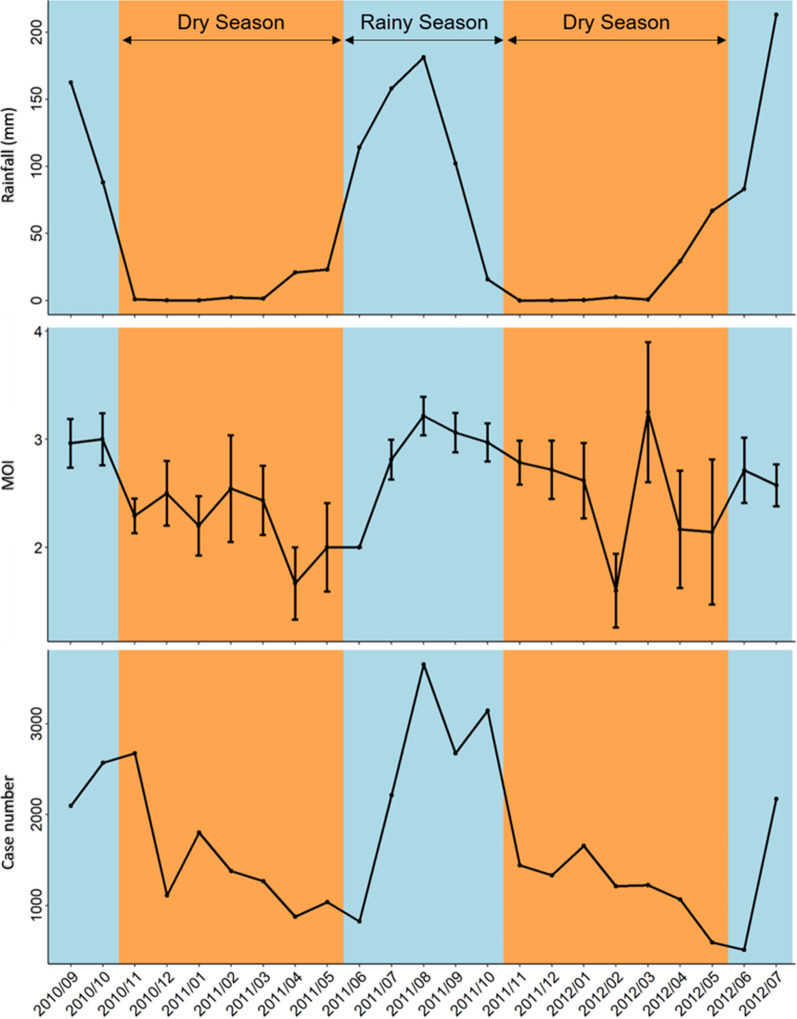
Monthly trend of rainfall, MOI, and malaria incidence in Nanoro area from September 2010 to July 2012. Rainy season is depicted in blue the dry season in orange. The upper panel shows the monthly trend of rainfall in the area (in mm) (source: GPM program). The middle panel illustrates the monthly trend of the average multiplicity of infection (MOI) ± SE, defined as the number of different parasite genotypes co-existing within a particular infection. The lower panel represents the reported total number of malaria cases per month during the study period (source: DHIS2-Endos medical data of the Center-West sanitary regional direction)

The monthly trends of rainfall, MOI, and malaria incidence in the study area are shown in Fig. 1. The dry season was characterized by lower MOI with a significant reduction of malaria cases. The peak MOI coincided with the malaria peak and the period of maximum rainfall (June to November) (Fig. 1).

**Fig. 2:**
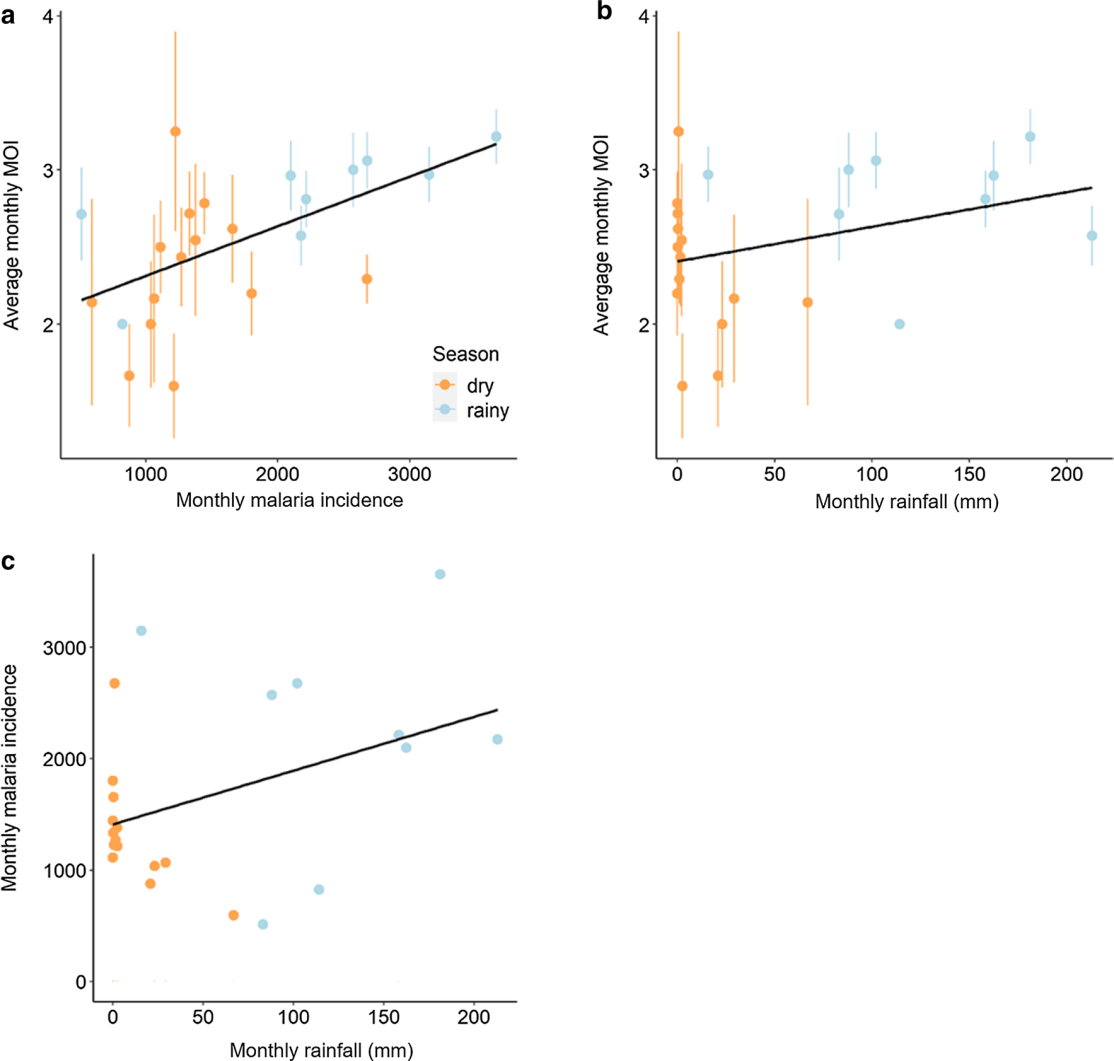
Relationship between the MOI, monthly malaria incidence and rainfall. **a** Correlation between the multiplicity of infection (MOI) defined as the number of different parasite genotypes co-existing within a particular infection and monthly malaria incidence (number of reported cases from DHIS2-Endos medical data of the Center-West sanitary regional direction). **b** Correlation between MOI and monthly rainfall (in mm) (source: GPM program. **c** Association between monthly malaria incidence and monthly rainfall (mm). Each point (blue, rainy season; orange, dry season) represents a value for malaria incidence. The line represents a linear relationship fitted to the number of malaria cases

There was a significant correlation between the monthly MOI and the monthly malaria incidence (*F*_(1, 21)_ = 11, *P* = 0.004) (Fig. 2a). However, monthly MOI and rainfall were poorly associated (*F*_(1, 21)_ = 2.6, *P* = 0.12) (Fig. 2b) as were malaria incidence and rainfall (*F*_(1, 21)_ = 3.9, *P* = 0.06) (Fig. 2c).

### Effect of the season, age and parasite density on the MOI

**Fig. 3:**
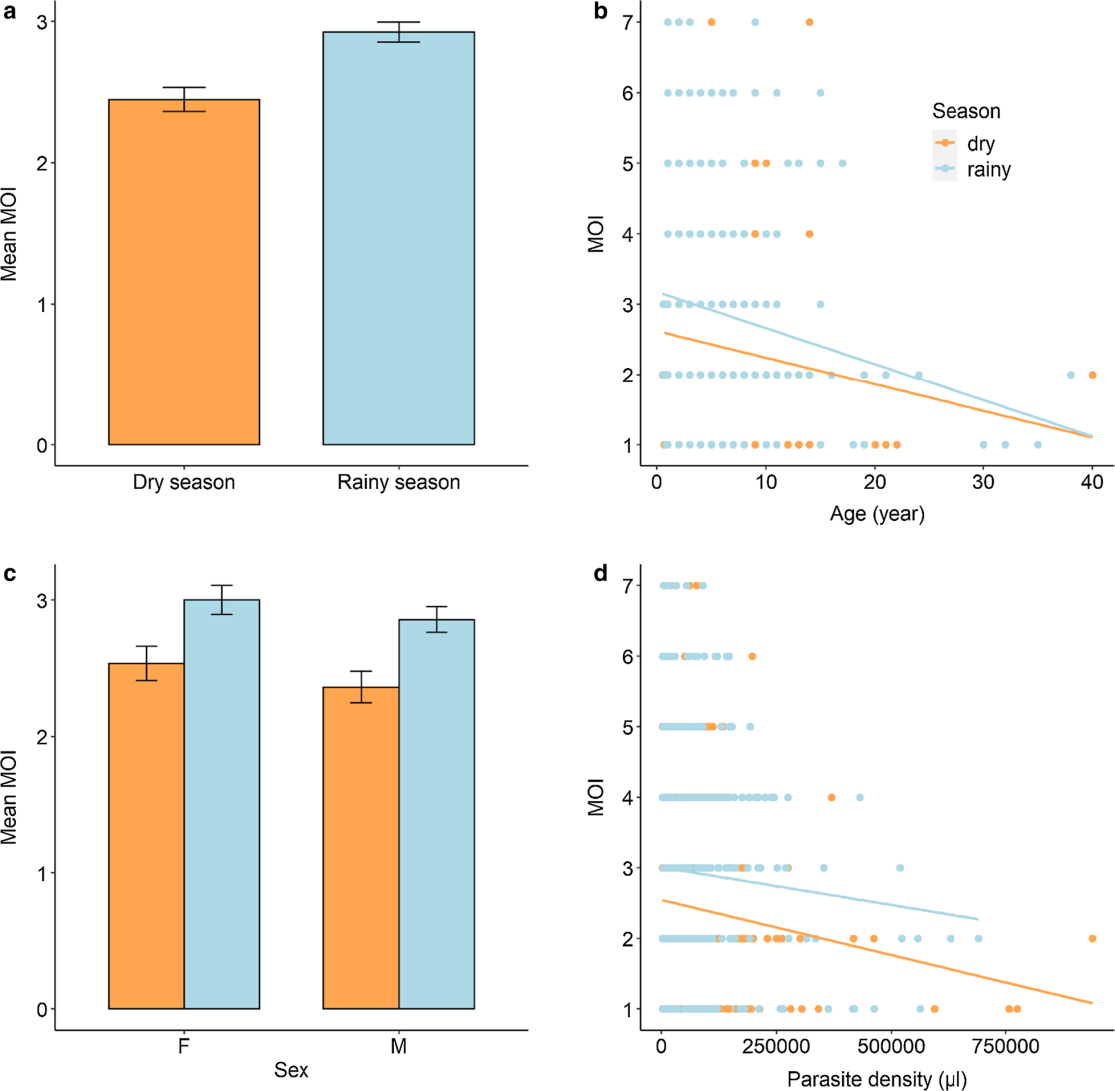
Relationship between the MOI, host age and sex, and parasite density. **a** Effect of season on multiplicity of infection (MOI), defined as the number of different parasite genotypes co-existing within a particular infection. Each color bar represents the average MOI for each season. **b** Effect of host age (in years) on MOI. Each color line represents a linear relationship fitted to the MOI values for each season (blue, rainy; orange = dry season). **c** Effect of host sex on MOI. Each color bar represents the average MOI for each sex by season (blue, rainy season; orange, dry season). **d** Effect of parasite density (number of asexual forms per microliter of blood) on MOI. Each color line represents a linear relationship fitted to the MOI values for each season (blue, rainy season; orange, dry season)

There was a strong effect of season on MOI (LRT $\chi_{1}^{2}$ = 15.74, *P* < 0.001), with infected patients harboring a higher number of parasite genotypes during the rainy season, rather than during the dry season (Fig. 3, Additional file 1: Table S1). MOI significantly decreased in older hosts (LRT $\chi_{1}^{2}$ = 14, *P* < 0.001) (Fig. 3b), regardless of the season (i.e. no statistical interaction between season and age, Additional file 1: Table S1). By excluding the few patients > 20 years-old (*n* = 11), the negative relationship between MOI and host age was confirmed (LRT $\chi_{1}^{2}$ = 7, *P* = 0.009). There was no significant sex effect on MOI (LRT $\chi_{1}^{2}$ = 1.36, *P* = 0.24) (Fig. 3c), with females and males harbouring an average 2.81 ± 0.08 and 2.66 ± 0.07 genotypes respectively. Finally, there was a negative relationship between MOI and parasite density (LRT $\chi_{1}^{2}$ = 8.1, *P* = 0.004) (Fig. 3d).

### Genetic diversity

From September 2010 to July 2012, the K1 allelic family was the most frequent *msp1* genetic variant circulating in infected patients ($\chi_{2}^{2}$ = 116, *P* < 0.001). Of a total of 1717 observed fragments for *msp1*, 879 (51%) belonged to the K1 family, 456 (27%) to MAD20 and 382 (22%) to RO33. The 3D7 allelic family was more frequent than the FC27 family ($\chi_{1}^{2}$ = 1, *P* < 0.001) with 58% (957/1653) of the *msp2* genetic variants.

### Monthly dynamic of the genetic composition of *msp1* and *msp2* allelic families

**Fig. 4:**
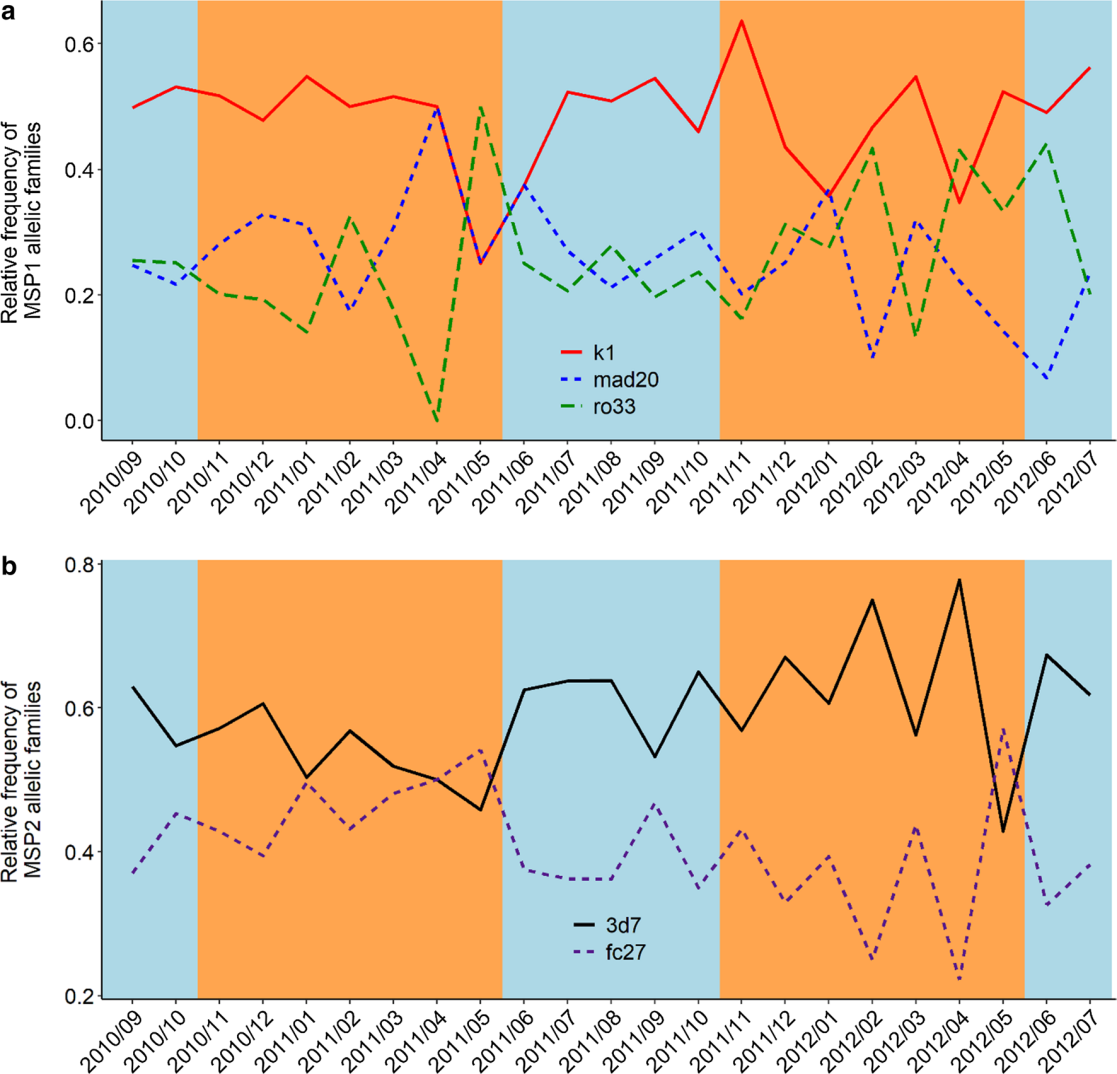
Temporal trend of *msp1* and *msp2* allelic families. **a** The temporal trend of *msp1* allelic families. Each color line represents the relative frequency level for each allelic family. **b** The temporal trend of *msp2* allelic families. Each color line represents the relative frequency level for each allelic family

Each allelic family of the *msp1* gene was present all year round. There was no significant monthly fluctuation of the *msp1* allelic families from September 2010 to July 2012 (K1: LRT $\chi_{1}^{2}$ = 0.007, *P* = 0.94, MAD20: LRT $\chi_{1}^{2}$ = 3, *P* = 0.085, LRT $\chi_{1}^{2}$ = 3, *P* = 0.083), (Fig. 4a). Similarly, there was no significant monthly fluctuation in the relative frequency of 3D7 and FC27 allelic families (LRT $\chi_{1}^{2}$ = 2.28, *P* = 0.13 for both family). However, toward the end of the dry season (May 2011 and May 2012), the over representation of K1 and 3D7 respectively, turns to diminish.

### Effects of season, patient age, sex and parasite density on genetic composition

**Fig. 5:**
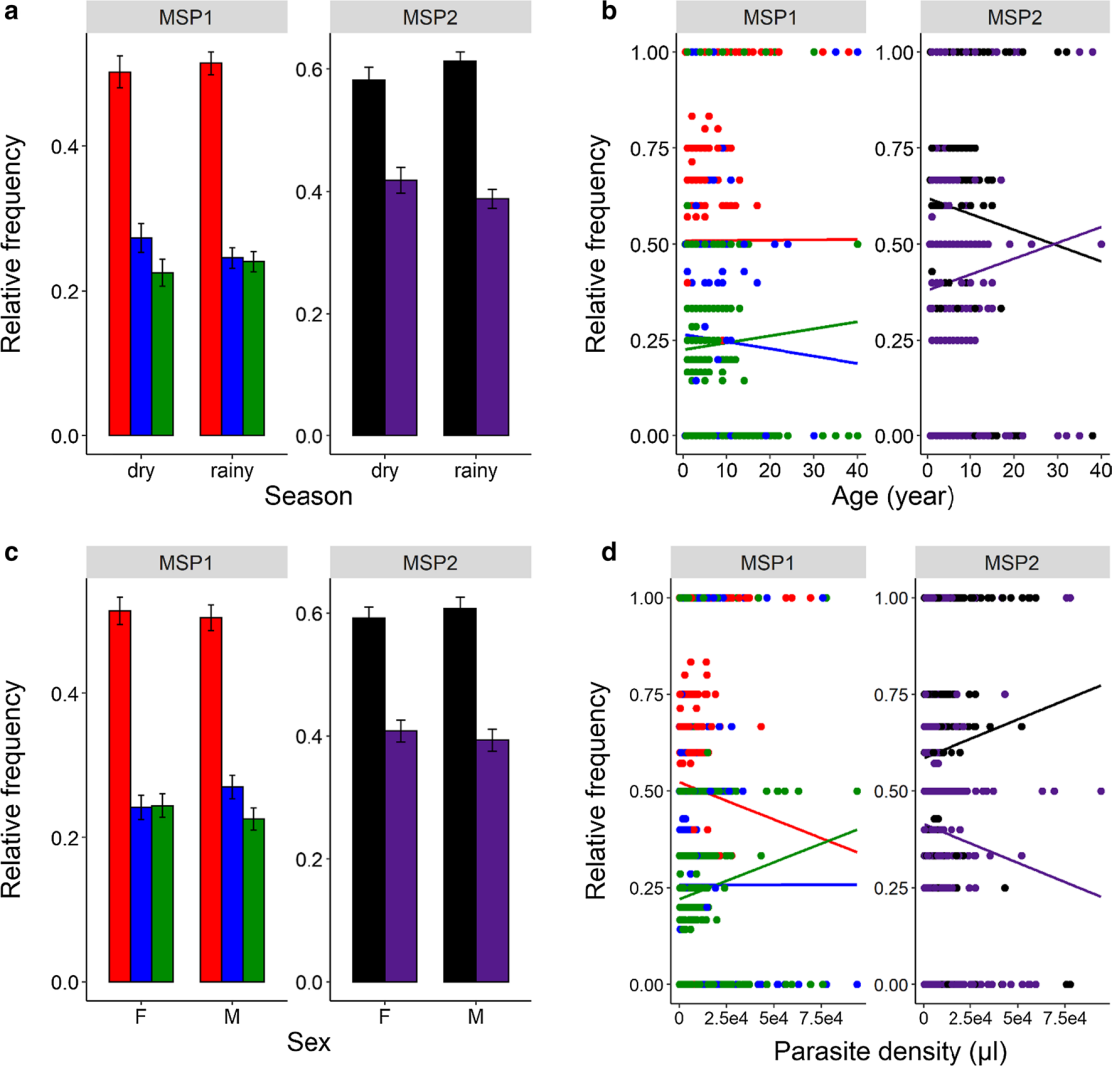
Influence of season, patient age, sex and parasite density on the relative frequencies of *msp1* and *msp2* allelic families. **a** Effect of season on the relative frequencies of *msp1* and *msp2* allelic families. Each color bar represents the relative frequency for each allelic family by season. **b** Effect of host age (in years) on the relative frequencies of *msp1* and *msp2* allelic families. Each color line represents a linear relationship fitted to the relative frequency level for each allelic family. **c** Effect of host sex on the relative frequencies of *msp1* and *msp2* allelic families. Each color bar represents the relative frequency for each allelic family by sex. **d** Effect of parasite density on the relative frequencies of *msp1* and *msp2* allelic families. Each color line represents a linear relationship fitted to the relative frequency level for each allelic family

**Fig. 6:**
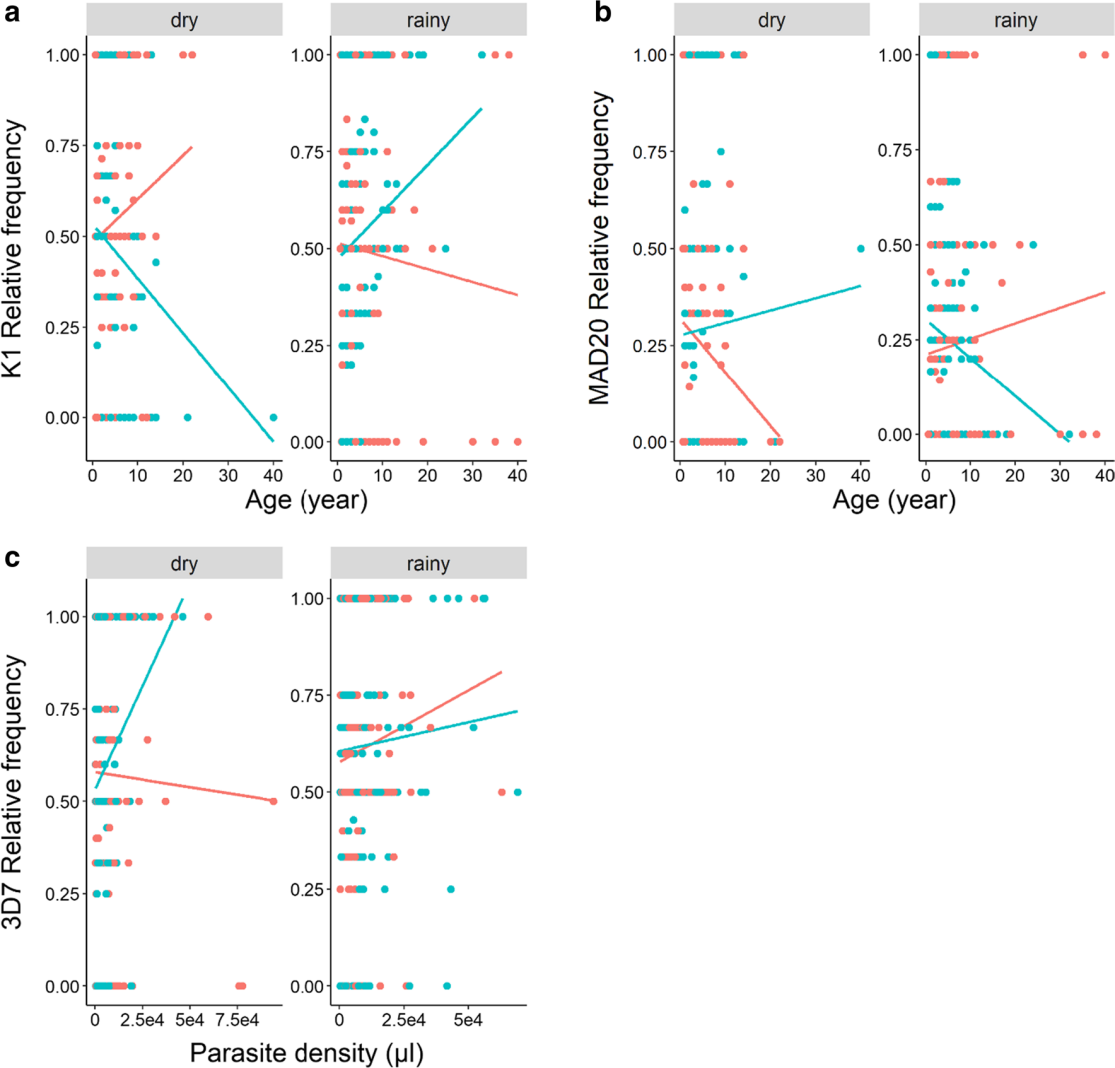
Three-way interaction between sex-season-patient age and sex-season-parasite density on the relative frequencies of *msp1* and *msp2* allelic families. **a** Effect of age on the relative frequency of *msp1*-K1 allelic family. Each color line represents a linear relationship fitted to the relative frequency level for each sex. **b** Effect of age on the relative frequency of *msp1*-MAD20 allelic family. Each color line represents a linear relationship fitted to the relative frequency level for each sex. **c** Effect of parasite density on the relative frequency of *msp2*-3D7 allelic family. Each color line represents a linear relationship fitted to the relative frequency level for each sex

The allelic family K1 was more frequent than the MAD20 and RO33 families regardless of the season (Fig. 5a, Additional file 2: Table S2). In addition, there were no main effects of patient age, sex or parasite density on K1 and MAD20 frequencies (Fig. 5a, Additional file 2: Table S2 and Additional file 3: Table S3). However, there was a significant three-way interaction between sex, season and patient age (Additional file 2: Table S2 and Additional file 3: Table S3). In particular, the K1 to MAD20 ratio increased with age in male patients during the rainy season but decreased during the dry season (Fig. 6a, b). The opposite pattern was observed for females (Fig. 6a, b). Finally, the relative frequency of RO33 variants increased in high density infections (LRT $\chi_{1}^{2}$ = 4.2, *P* = 0.04).

The allelic family 3D7 was more frequent than the FC27 family regardless of the season (Fig. 5a, Additional file 5: Table S5 and Additional file 6: Table S6). There were no main effects of season, patient age and sex on the relative frequency of 3D7 and FC27 (Fig. 5, Additional file 5: Table S5 and Additional file 6: Table S6). However, there was a significant three-way interaction between sex, season and parasite density (Additional file 5: Table S5 and Additional file 6: Table S6). In particular, the relative frequency of 3D7 during the dry season decreased with parasite density in female patient only (Fig. 6c).

## Discussion

From the start of the study (2010) to the end (2012) no significant difference in MOI values was observed from year to year. The study started just after the large-scale distribution of insecticide-treated nets (ITN) and no major intervention occurred during the course of the study, probably explaining the lack of significant MOI variation between the beginning in 2010 and the end of the study in 2012. The high MOI value observed in this study indicates the endemicity level in the area while highlighting that the expected outcome from the large-scale deployment of ITN was not achieved. High MOI results from high diversity in *P. falciparum* population structure observed in high transmission settings while low transmission settings are characterized by a predominance of monoclonal infections [28–30]. The MOI level in Nanoro area is comparable to previous findings in the country and neighboring countries across West Africa with similar level of transmission intensity [19, 31–36]. However, the reported MOI values would be significantly underestimated due to genotyping strategy as this study used agarose gel approach to detect parasite variants *versus* new methods with higher discriminatory power such as capillary electrophoresis and genome-wide association studies and this represents one limitation of this study.

A match between the peak MOI and malaria peak and the period of maximum rainfall was observed, suggesting that MOI could be a reliable indicator describing malaria transmission intensity in high endemic malaria settings as previously suggested [5]. However, in reduced malaria settings, previous reports showed that MOI is a poor predictor of malaria transmission intensity [37].

Our study reported a strong effect of season on the multiplicity of *P. falciparum* infections. In contrast to studies in Ghana where an association between highest MOI with low transmission season was reported [19, 34], our study found higher MOI values in the rainy season than in the dry season reflecting the known variation in seasons of malaria transmission in Burkina Faso. This could be due to the differences in population profiles. The studies in Ghana were actually carried out on asymptomatic populations with increased MOI values compared to symptomatic populations on whom the present work is based. Unlike this study, no seasonal fluctuation of the MOI was reported in the Democratic Republic of the Congo [38] highlighting the geographical differences in the expression of the MOI. That is probably why the MOI is not a reliable indicator of malaria transmission in areas with reduced intensity of malaria transmission [37].

A negative correlation between the MOI and parasite density was observed, suggesting a within-host competition among co-infecting genetically distinct *P. falciparum* variants. This competition becomes clearly apparent when analyzing the interactions of different allelic families in mixed infections as previously reported [14]. This finding partially explains why the MOI is higher in asymptomatic populations (with low parasite load) than in symptomatic populations as previously pointed out by several authors [39–41]. This means that clinical manifestations result from a breakdown of the balance among genetically distinct variants (from asymptomatic infections) leading to proliferation of more competitive variants over the others. Likewise, it has been reported previously that multiclonal asymptomatic infections reduced the risk of malaria disease [42].

Similarly, a strong effect of host age on MOI was observed presumably because of the acquisition of antiparasitic immunity in high malaria endemic settings [10, 34, 43, 44].

A high diversity of circulating *P. falciparum* variants was found in the Nanoro area. All allelic families of *msp1* and *msp2* genes were found in both the dry and the rainy season. The absence of association between particular *msp1* or *msp2* alleles and a particular season was previously reported [8, 34]. However, the *msp1*-K1 and *msp2*-3D7 allelic families predominated over the others regardless of season. The predominance of the *msp1*-K1 and *msp2*-3D7 was reported by previous findings in Burkina Faso [31, 32]. This could mean that these two allelic families are better adapted to their host in the study area than the others and could be the reason why they are less virulent compare to the MAD20, RO33 and FC27 allelic families which are mostly associated with hyper-parasitaemia and anemia respectively [14], as well as the increased probability of clinical malaria occurrence [45]. The relative frequency of RO33 variants increased in high density infections, suggesting that these variants suffer more from within host competition than the K1 and MAD20 variants. Surprisingly, a three-way interaction between host-age, sex, season on the relative frequencies of *P. falciparum* genetic variants was observed. The K1 to MAD20 ratio increased with age in male patients during the rainy season but decreased during the dry season. Similarly, the relative frequency of 3D7 during the dry season decreased with parasite density in female patients only. These findings highlight the multifactorial aspect underlying the development of a particular variant within the host, ranging from genetic (parasite: effect observed with specific variant and not with the others; host: effect observed with particular sex and not with the other) to environmental factors. As the majority of the study population is represented by children under five years-old, no sex-related behavior differences in each season could explain these findings. However, hormonal and immunological mechanisms mediating sex differences in parasite infection has been suggested [16, 17], highlighting the need for further investigation on host related patterns.

## Conclusions

In high malaria endemic settings with marked variation in seasons of malaria transmission, MOI represents an appropriate malaria metric which provides useful information about the changes in malaria transmission longitudinally in a given area. All allelic families of *msp1* and *msp2* genes were found in both dry and rainy season. However, patterns affecting the distribution of these *P. falciparum* variants deserve further investigations. The approach offers the opportunity of translating genotyping data into relevant epidemiological information for malaria control.

## Supplementary information

**Additional file 1: Table S1.** Determinants of the multiplicity of infection (MOI). Effect of season, patient age, sex, parasite density, and interactions on the MOI. Significant effects are emphasized in bold.

**Additional file 2: Table S2.** Determinants of the frequency of the K1 allelic family. Effect of season, patient age, sex, parasite density, and interactions on the proportion of K1 variants in infected patient. Significant effects are emphasized in bold.

**Additional file 3: Table S3.** Determinants of the frequency of the MAD20 allelic family. Effect of season, patient age, sex, parasite density, and interactions on the proportion of MAD20 variants in infected patient. Significant effects are emphasized in bold.

**Additional file 4: Table S4.** Determinants of the frequency of the RO33 allelic family. Effect of season, patient age, sex, parasite density, and interactions on the proportion of RO33 variants in infected patient. Significant effects are emphasized in bold.

**Additional file 5: Table S5.** Determinants of the frequency of the 3D7 allelic family. Effect of season, patient age, sex, parasite density, and interactions on the proportion of 3D7 variants in infected patient. Significant effects are emphasized in bold.

**Additional file 6: Table S6.** Determinants of the frequency of the FC27 allelic family. Effect of season, patient age, sex, parasite density, and interactions on the proportion of FC27 variants in infected patient. Significant effects are emphasized in bold.

## Data Availability

Data supporting the conclusions of this article are included within the article and its additional files.

## Abbreviations

AL: Artemether-lumefantrine
ASAQ: Artesunate-amodiaquine
DNA: Deoxyribonucleic acid
DHIS2: District Health Information Software 2
GLM: Generalized linear model
GPM: Global precipitation measurement
ITN: Insecticide-treated nets
LRT: Likelihood ratio test
MOI: Multiplicity of infection
MSP1: Merozoite surface protein 1
MSP2: Merozoite surface protein 2
PCR: Polymerase chain reaction
