# Supplementary material for: Determinants of Plasmodium falciparum multiplicity of infection and genetic diversity in Burkina Faso
Source: Parasit Vectors. 2020 Aug 20;13:427. doi: 10.1186/s13071-020-04302-z (PMC7441709; doi:10.1186/s13071-020-04302-z)
Supplement: Supplementary file 1 — Additional file 1: Table S1. Determinants of the multiplicity of infection (MOI). Effect of season, patient age, sex, parasite density, and interactions on the MOI. Significant effects are emphasized in bold. [file 13071_2020_4302_MOESM1_ESM.docx]

**Additional file 1**

**Determinants of *Plasmodium falciparum* multiplicity of infection and genetic diversity in Burkina Faso**

Paul Sondo^1*^, Karim Derra^1^, Toussaint Rouamba^1^, Seydou Diallo-Nakanabo^2^, Paul Taconet^3^ Adama Kazienga^1^, Hamidou Ilboudo^1^, Marc-Christian Tahita^1^, Innocent Valéa^1^, Herman Sorgho^1^, Thierry Lefèvre^3,4,5^ and Halidou Tinto^1^

^1^Institut de Recherche en Sciences de la Santé/Clinical Research Unit of Nanoro (IRSS-URCN), Nanoro, Burkina Faso.

^2^Institut National de Santé Publique/Centre Muraz de Bobo-Dioulasso, Bobo-Dioulasso, Burkina Faso.

^3^Centre de Recherche en Écologie et Évolution de la Santé (CREES), Montpellier, France.

^4^Laboratoire Mixte International sur les Vecteurs (LAMIVECT), Bobo Dioulasso, Burkina Faso.

^5^Maladies Infectieuses et Vecteurs: Ecologie, Génétique, Evolution et Contrôle (MIVEGEC), Université de Montpellier, Institut de Recherche pour le Développement (IRD), Centre National pour la Recherche Scientifique (CNRS), Montpellier, France.

**Table S1: Determinants of the Multiplicity Of Infection (*MOI*)**: Effect of season, patient age, sex, parasite density, and interactions on the *MOI*. Significant effects are emphasized in bold.
