# Supplementary material for: Determinants of Plasmodium falciparum multiplicity of infection and genetic diversity in Burkina Faso
Source: Parasit Vectors. 2020 Aug 20;13:427. doi: 10.1186/s13071-020-04302-z (PMC7441709; doi:10.1186/s13071-020-04302-z)
Supplement: Supplementary file 5 — Additional file 5: Table S5. Determinants of the frequency of the 3D7 allelic family. Effect of season, patient age, sex, parasite density, and interactions on the proportion of 3D7 variants in infected patient. Significant effects are emphasized in bold. [file 13071_2020_4302_MOESM5_ESM.docx]

**Additional file 5**

**Determinants of *Plasmodium falciparum* multiplicity of infection and genetic diversity in Burkina Faso**

Paul Sondo^1*^, Karim Derra^1^, Toussaint Rouamba^1^, Seydou Diallo-Nakanabo^2^, Paul Taconet^3^ Adama Kazienga^1^, Hamidou Ilboudo^1^, Marc-Christian Tahita^1^, Innocent Valéa^1^, Herman Sorgho^1^, Thierry Lefèvre^3,4,5^ and Halidou Tinto^1^

^1^Institut de Recherche en Sciences de la Santé/Clinical Research Unit of Nanoro (IRSS-URCN), Nanoro, Burkina Faso.

^2^Institut National de Santé Publique/Centre Muraz de Bobo-Dioulasso, Bobo-Dioulasso, Burkina Faso.

^3^Centre de Recherche en Écologie et Évolution de la Santé (CREES), Montpellier, France.

^4^Laboratoire Mixte International sur les Vecteurs (LAMIVECT), Bobo Dioulasso, Burkina Faso.

^5^Maladies Infectieuses et Vecteurs: Ecologie, Génétique, Evolution et Contrôle (MIVEGEC), Université de Montpellier, Institut de Recherche pour le Développement (IRD), Centre National pour la Recherche Scientifique (CNRS), Montpellier, France.

**Table S5: Determinants of the frequency of the 3D7 allelic family**: Effect of season, patient age, sex, parasite density, and interactions on the proportion of 3D7 variants in infected patient. Significant effects are emphasized in bold.

| Variables | Df | LRT *X^2^_1_* | P-value |
| --- | --- | --- | --- |
| Season | 1 | 1.1e+00 | 0.295 |
| Density | 1 | 2.5e+00 | 0.117 |
| Age | 1 | 5.8e-01 | 0.445 |
| Sex | 1 | 5.9e-01 | 0.442 |
| Season:Density | 1 | 2.1e-01 | 0.647 |
| Season:Age | 1 | 3.7e-01 | 0.546 |
| Density:Age | 1 | 2.0e-01 | 0.654 |
| Season:Sex | 1 | 2.9e-01 | 0.590 |
| Density:Sex | 1 | 4.0e-02 | 0.842 |
| Age:Sex | 1 | 3.9e-02 | 0.843 |
| Season:Density:Age | 1 | 2.3e+00 | 0.127 |
| Season:Density:Sex | **1** | **4.1e+00** | **0.042 *** |
| Season:Age:Sex | 1 | 6.6e-05 | 0.994 |
| Density:Age:Sex | 1 | 4.1e-01 | 0.522 |
| Season:Density:Age:Sex | 1 | 1.3e-01 | 0.717 |
